# Barriers that limit the implementation of thermal fogging for the control of dengue in Colombia: a study of mixed methods

**DOI:** 10.1186/s12889-019-7029-1

**Published:** 2019-05-30

**Authors:** Andrés F. Usuga, Lina M. Zuluaga-Idárraga, Natalia Alvarez, Raúl Rojo, Enrique Henao, Guillermo L. Rúa-Uribe

**Affiliations:** 1 Grupo Epidemiología, Linea enfermedades infecciosas, Facultad Nacional de Salud Pública, Universidad de Antioquia, Calle 62 # 52-60, Antioquia Medellin, Colombia; 2 Grupo Entomología Médica, Facultad de Medicina, Universidad de Antioquia, Carrera 51d # 62-29 Lab. 303, Antioquia Medellin, Colombia; 3 Grupo Salud y Ambiente, Facultad Nacional de Salud Pública, Universidad de Antioquia, Calle 62 # 52-59, Antioquia Medellin, Colombia; 4 Grupo de Investigación Diverser, Facultad de Educación, Universidad de Antioquia, Calle 62 # 52-59, Antioquia Medellin, Colombia; 5 Secretaría de Salud de Medellin, Carrera 53ª # 42-101 piso 10, Antioquia Medellin, Colombia

**Keywords:** Dengue, *Aedes aegypti*, Fumigation, Thermal fogging, Acceptability, Implementation research

## Abstract

**Background:**

Thermal fogging of Insecticides is a vector control strategy used by the Medellin Secretary of Health to combat dengue. This method is employed during outbreaks to curb populations of potentially infectious adult mosquitoes and interrupt transmission cycles. While this strategy has been used in Medellin since 2007, in some years it has not reduced dengue cases as expected. Difficulties in the implementation of fumigation strategies, such as lack of opportunity for treatment and public perception may be factors that limit its utility. The objective of this study was to identify barriers that hinder the implementation of thermal fogging, as well as attitudes and beliefs that prevent its acceptance.

**Methods:**

We used a cross-sectional observational study of mixed methods carried out in neighborhoods prioritized for fumigation treatment in Medellin, Colombia. First, we assessed the timeliness of treatment by determining the latency period between reported dengue cases and the implementation of fumigation in response to those cases. Next, we administered structured questionnaires to residents in the area of fumigation treatments (*n* = 4455 homes) to quantify acceptance and rejection, as well as factors associated with rejection.

**Results:**

The median time between notification and treatment was 25 days (IQR 20.0–36.5). Fumigators were only able to treat 53.7% of total households scheduled for treatment; 9.6% rejected treatment, and treatment teams were unable to fumigate the remaining 36.7% of homes due to absent residents, no adults being present, and other reasons. The most frequent causes for rejection were residents being busy at the time of treatment (33.1%) and no interest in the treatment (24.5%). Other reasons for rejection include the perceptions that fumigation does not control pests other than mosquitoes (4.3%), that no mosquitoes were present in the home (3.3%), and that fumigation affects human health (3.1%).

**Conclusions:**

The high percentage of houses where it was not possible to perform fumigation limits control of the vector. Future strategies should consider more flexible treatment schedules and incorporate informational messages to educate residents about the safety and importance of treatment.

**Electronic supplementary material:**

The online version of this article (10.1186/s12889-019-7029-1) contains supplementary material, which is available to authorized users.

## Background

Dengue fever has been the most widespread arboviral disease in the tropics and subtropics during the last three decades [1]. The World Health Organization (WHO) estimates that 3.9 billion people in 128 countries are at risk of contracting a dengue infection, primarily in Asia, Africa and Latin America [2]. In Latin America, reported cases of dengue have increased dramatically over the last two decades [3]. Dengue virus transmission has been reported in all countries in this region except Chile and Uruguay [4]. According to the WHO [5], the American countries with the highest number of cases reported are Brazil, Mexico and Colombia. Particularly in the latter, in 2017 there were 26,279 cases, of which 286 were severe dengue, resulting in 60 deaths. The incidence in Colombia was estimated at 93 cases per 100,000 inhabitants for the year 2017 [6]. For Medellin, the second most populous city in the country, the number of documented cases represents about 8.3% of the national total [6].

The mitigation of outbreaks and prevention of dengue diseases in cities like Medellin depend largely on vector control. Ministries of Health have many approaches and tools at their disposal to prevent and control dengue, the probability of success of each method depends on several factors. Some of these factors are the geographic coverage, frequency of application and acceptance of the interventions by the target populations [7].

To control dengue, the WHO recommends the application of insecticides as part of the Integrated Management Strategy for Dengue Prevention and Control (IMS-Dengue) [8]. In Medellin, the Secretary of Health (Secretaria de Salud de Medellin; SSM) has used thermal fogging periodically since 2007 [9]. This treatment involves the aerosolization and dispersion of an insecticide to cause the immediate death of adult mosquito populations [8, 10]. The entomological efficacy of this treatment for dengue has been widely demonstrated in different studies, obtaining vector mortalities from 70.0 to 90.0% [11–13]. The SSM uses malathion (an organophosphate) as the active substance in its thermal fogging program [14].

Despite the entomological efficacy of fumigation, new cases of dengue are sometimes reported in areas that received treatment [6, 15], and the epidemiological impact of thermal fogging (that is, its ability to reduce dengue transmission) remains unclear [16]. Limited success in reducing cases of dengue could be due to barriers that lead to sub-optimal coverage, such as lack of accessibility to houses [17] or rejection by residents due to safety concerns [18]. According to the recommendations of some ministries of health, coverage must be at least 95.0% in order to substantially reduce dengue cases [19]. It has been observed for other vectors that low levels of acceptance may allow surviving individuals to recover in untreated dwellings and re-colonize homes which have already received treatment [20].

In addition to the opportunity and coverage of the treatment, some studies have identified several social determinants for the rejection of fumigation, such as: limited knowledge of the target disease, concerns over the risk of becoming ill, communication failures on the part of health officials, and perception of poor efficacy of fumigation [21]. Another barrier to the effective implementation of thermal fogging is the timing of the treatment; when treating an area based on a focal case, the risk of dengue transmission in intimately associated with the extrinsic incubation period (that is, the time necessary for the mosquito to become infectious after having bitten a viremic host) [22, 23]. The extrinsic incubation period reported for dengue is about 9 days (IQR 6–17 d) and is highly dependent on the temperature [22]. For this reason, vector control strategies should be implemented in the shortest possible time after notification of the case [1, 24]. The objective of this study was to improve the epidemiological efficacy of fumigation by identifying opportunity and acceptability barriers that limit its implementation in Medellin.

## Methods

In order to assess barriers to the implementation of thermal fogging, we performed three analyses. First, we analyzed data regarding the timing of dengue cases and preventive thermal fogging around transmission foci from January to June 2016. Next, we administered structured questionnaires to households targeted for treatment. Finally, we hosted two focus groups discussions in August 2016.

### Study design

A design of mixed methods with an explanatory sequential type was used, with both quantitative and qualitative phases. The quantitative phase was conducted with a cross-sectional observational design, with elements of case-control study, and the qualitative elements of grounded theory were employed using focus groups discussion (FGD) [25].

### Location of the study

The study was conducted in Medellin, Colombia (6 ° 14 ‘41.09 “N, 75 ° 34’ 29.38” W), located at 1475 m above sea level, with an average annual temperature of 24 °C and precipitation of 1571 mm/year [26]. The city is classified as hyperendemic for dengue, where neighborhoods with high rates of incidence of the disease and active transmission centers are prioritized by the SSM to receive treatment with thermal fogging [21]. Neighborhoods in this study were not selected randomly throughout the city due to the heterogeneous distribution of dengue foci in Medellin. Thus, while questionnaire responses do not represent a random sample of residents in the city, they nonetheless capture the perceptions of the most at risk residents.

### Quantitative phase

#### Treatment opportunity

To determine the timing of the treatment, all records of dengue cases reported to the Epidemiological Surveillance System (SIVIGILA) in Medellin were analyzed for the period of January 01 to June 27, 2017. Variables included were the dates of: symptoms onset, consultation, notification, and treatment with fumigation. All fumigation timelines without missing data points were included in analysis (*n* = 64).

#### Willingness to accept treatment

The acceptance and rejection of treatment was evaluated in two phases: quantification of acceptance and rejection, and exploration of factors associated with each. To quantify acceptance and rejection rates, SSM officials conducting fumigation in dengue-positive neighborhoods recorded whether each home’s owner accepted fumigation, rejected treatment, or whether it was not possible to fumigate (e.g., no adult was present, or pets could not feasibly be removed from the home). The reason for not treating homes in the latter category was also recorded. In total, 4455 homes were visited from May to June 2016.

To explore the factors associated with rejection, written questionnaires were administered to a subset of all homes visited. Homes were sampled following a case-control design. A sample size of 200 homes that accepted and 200 homes that rejected treatment was estimated based on an odds ratio (OR) of 1.8 (confidence interval = 95%, power = 80%). Subjects who accepted fumigation were chosen via simple random sampling, whereas subjects who rejected treatment consisted all of the people who rejected fumigation. Both groups received a structured questionnaire of 38 questions about personal information, perceptions of thermal fogging, knowledge of dengue and mosquitoes, practices of mosquito prevention, and housing information (Additional file 1). To explore the association between rejection and sociodemographic factors, perceptions and knowledge, crude and adjusted OR were calculated with their respective 95% confidence intervals, using a logistic regression model. Variables were included in the model based on Hosmer-Lemeshow criteria (*p* < 0.25). In addition, several variables were chosen a priori to be included based on their likely relevance according to a literature search. All analyses were carried out in Stata [27] (Stata Statistical Software: Release 15, College Station, TX, USA).

#### Qualitative phase

We used FGD to qualitatively understand willingness and rejection to accept fumigation [25, 28]. We invited 30 people that previously responded to the questionnaire, with 15 that accepted the treatment and 15 that rejected it. Unfortunately, just eight people who accepted and four people who rejected the treatment attended FGDs. Participants were allocated into two groups: one with those that rejected treatment and one with those that accepted it. Participants invited to participate in FGDs were selected based on their ability to express their thoughts and opinions clearly, as observed by the teams administering questionnaires. The unit of analysis was the group, making use of the collective narrative, while the units of study were the people who participated in each FGD [25].

To analyze the qualitative information, there were three categories and seven subcategories, chosen from the reviewed literature. The categories included were. i. Experiences: events that do not depend on the subject but that occur in the subject, form it and transform it, ii. Meanings: conception that the subject makes of the phenomena that are presented to him or her, and iii. Reasons: justification of the behavior [29–31]. Categories were tracked within the discourse, and emerging issues such as planning of land use and perceptions of SSM were identified. The analysis of qualitative data was carried out with the Atlas.ti [32].

## Results

### Opportunity of treatment

Between January 1 and June 27, 2017, 1568 probable cases of dengue were registered in Medellin, of which 64 were chosen for treatment with fumigation by SSM based on spatiotemporal clustering. From these, the time frame for treatment with thermal fogging was analyzed (Additional file 2). In general, patients consulted health services quickly after the onset of symptoms (Median 3 days, IQR: 1.0–5.0), and the relay of positive cases to the epidemiological surveillance system was also fast (median 6 days, IQR 3.0–8.5). However, time from notification to treatment with thermal fogging was usually greater than 3 weeks (Table 1).

**Table 1:** Time elapsed between the onset of symptoms, consultation, notification and treatment with fumigation

Variable	Median (IQR)
Days between onset of symptoms and doctor’s consultation	3 (1.0–5.0)
Days between onset of symptoms and notification of surveillance system	6 (3.0–8.5)
Days between onset of symptoms and fumigation	32 (25.0–40.0)
Days between notification of surveillance system and treatment	25 (20.0–36.5)

*IQR* Interquartile range

### Outcomes of requests to treat with thermal fogging

**Fig. 1:**
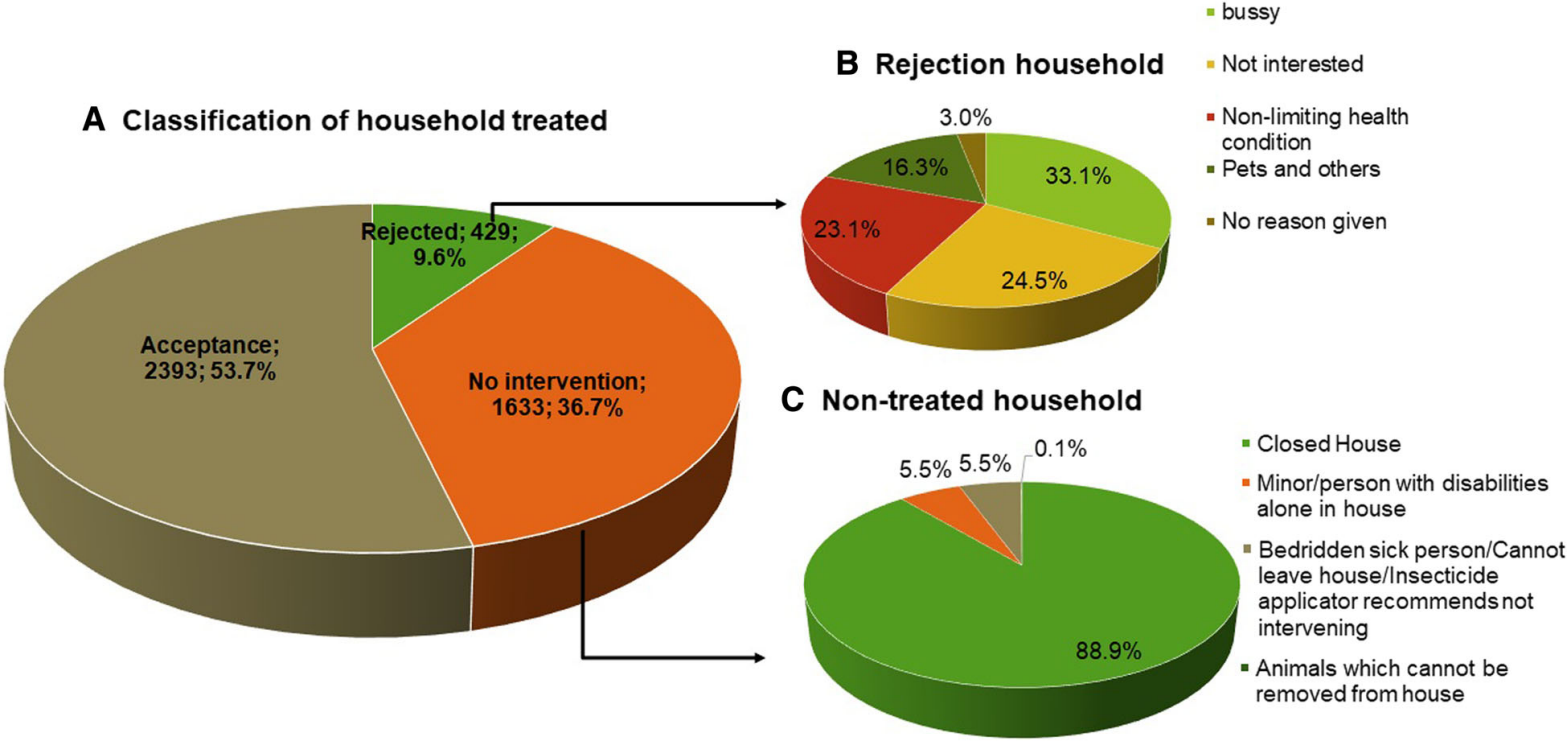
Distribution of dwellings included in the treatment areas (**a**), reasons that residents rejected treatment (**b**), and reasons that treatment of dwellings was impossible (**c**)

Of the 4455 dwellings included (Additional file 3), 2393 (53.71%) accepted the treatment, 429 (9.63%) rejected it and in 1633 (36.66%) it was not possible to perform fumigation (Fig. 1a). In reluctant dwellings with reluctant residents, the main reasons for not allowing the treatment was being busy (33.1%) or lack of interest in fumigation (24.5%) (Fig. 1b). No resident being present (88.9%) was the primary reason that fumigation was not possible (Fig. 1c).

### Rejection of fumigation

The sociodemographic characteristics of survey participants are presented in Table 2 to explore the factors associated with rejection. These sociodemographic characteristics were obtained from 410 residents that responded to the questionnaire (Additional file 3).

**Table 2:** Sociodemographic characteristics of the group of people who accepted or were reluctant about treatment with fumigation

	Acceptance *n* = 206	%	Rejection *n* = 204	%
Sociodemographic characteristics				
Sex; n (%)				
Women	159	77.2	156	76.5
Men	47	22.8	48	23.5
Occupation; n (%)				
Head of Household and/or housewife	159	77.2	143	70.1
Student	9	4.4	13	6.4
Informal	15	7.3	29	14.2
Unemployed	11	5.3	10	4.9
Retiree	12	5.8	9	4.4
Age in years; median (IQR)	49	37–62	51	34–62
Educational level; n (%)				
Elementary school or less	73	35.4	77	37.7
Any level of high school	103	50.0	88	43.1
Any level of higher education	30	14.6	38	18.7
Missing value	0	0.0	1	0.5
Believes that fumigation is beneficial; n (%)				
Yes	203	98.5	177	86.8
No	1	0.5	27	13.2
Missing value	2	1.0	0	0.0
Time lived in neighborhood (y); median (IQR)	25	11–40	20	7–36
Amount of time in house; n (%)				
All the time	174	84.5	158	77.5
Only in the mornings	11	5.3	19	9.3
Only in the afternoons	7	3.4	7	3.4
Only at night	14	6.8	20	9.9

A high percentage of the people surveyed consider that fumigation protects against dengue, eliminates mosquitoes and controls other insect pests. In addition, they recognize that the SSM performs it, it is free and they trust the officials who visit them. Despite this, percentages higher than 31% perceive that fumigation can affect human and pet health (Table 3).

**Table 3:** Perceptions and knowledge of fumigation treatment of the group of people who accepted or were reluctant to accept fumigation treatment

Fumigation (perception of people interviewed)	Acceptance *n* = 206	%	Rejection *n* = 204	%
It protects against dengue; n (%)				
Yes	190	92.2	164	80.4
No	7	3.4	30	14.7
Do not know	9	4.4	10	4.9
It kills mosquitoes; n (%)				
Yes	174	84.5	160	78.4
No	18	8.7	25	12.3
Do not know	14	6.8	19	9.3
It kills other pests in addition to mosquitoes; n (%)				
Yes	183	88.8	127	62.2
No	10	4.9	42	20.6
Do not know	13	6.3	35	17.2
It is free; n (%)				
Yes	200	97.1	189	92.6
No	2	1.0	2	1.0
Do not know	4	1.9	13	6.4
Trust for those conducting the fumigation; n (%)				
Yes	201	97.6	164	80.4
No	1	0.5	33	16.2
Do not know	4	1.9	7	3.4
It affects the health of people; n (%)				
No	126	61.2	61	29.9
Yes	64	31.1	118	57.8
Do not know	16	7.8	25	12.6
It affects the health of pets; n (%)				
No	72	35.0	50	24.5
Yes	112	54.4	122	59.8
Do not know	22	10.7	32	15.7
It damages furniture; n (%)				
No	195	94.7	178	87.3
Yes	1	0.5	12	5.9
Do not know	10	4.9	14	6.9
It causes bad smells; n (%)				
No	70	34.0	46	22.6
Yes	131	63.6	146	71.6
Do not know	5	2.4	12	5.9
It damages the environment; n (%)				
No	143	69.4	119	58.3
Yes	37	18.0	51	25.0
Do not know	26	12.6	34	16.7
The institution which performs the fumigation;				
n (%)				
SSM	165	80.1	141	69.1
Do not know	36	17.5	55	27.0
Other	4	1.90	7	3.4
Missing value	1	0.5	1	0.5
Previous treatments; n (%)				
Yes	164	79.6	132	64.7
No	41	19.9	63	30.9
Do not remember	1	0.5	8	3.9
Missing value	0	0.0	1	0.5

Five missing values were identified and removed from analysis. For the remaining 405 values the rejection of treatment was associated with the perception that it affects health (adjusted OR 3.0, 95% CI 1.9–4.9), no mosquitoes observed in the home (adjusted OR 3.3, 95% CI 1.9–5.9), not recognizing the symptoms of the disease (adjusted OR 1.6, 95% CI 1.0–2.6), the dwelling having been previously treated with thermal fogging (adjusted OR 2.4, 95% CI 1.1–3.2), and with the perception that fumigation does not control other pests besides mosquitoes (adjusted OR 4.5, 95% CI 3.0–8.6) (Table 4).

**Table 4:** Factors associated with rejection about fumigation

	OR	95% CI	Adjusted OR^a^	95% CI
It controls pests other than mosquitoes				
Yes				
No/Do not know	5.1	2.9–8.1	4.5	3.0–8.6
Mosquitoes in house				
Yes				
No	3.4	2.0–5.6	3.3	1.9–5.9
It affects the health of people				
No				
Yes	3.8	2.5–5.9	3.0	1.9–4.9
Do not know	3.2	1.6–6.5	2.8	1.3–6.1
The household has been treated with fumigation previously				
Yes				
No	2.1	1.4–3.4	2.4	1.1–3.2
Is aware of symptoms				
Yes				
No	1.5	1.0–2.3	1.6	1.0–2.6
It protects from Dengue				
Yes				
No /Do not know	3.1	1.6–5.8	1.9	0.9–3.9
It causes bad smells				
No				
Yes	1.7	1.1–2.7	1.1	0.7–1.9
Do not know	3.7	1.2–11.1	2.3	0.6–8.0

*OR* estimated by a logistic regression model

^a^ *Value adjusted by other variables included in the model*

### Experiences, meaning, and reasons related to the rejection of fumigation

Participants in the reluctant focus group discussion (RFGD) assert that mosquitoes are found outside their homes, in places such as streams, green areas and recycling sites, and mention that: “[SSM] should fumigate the stream because the quantity [of mosquitoes] is greater there, and in the corner of my house, there is a recycling site which is never fumigated” (RFGD). In addition, they tended to believe that fumigation does not reduce the densities of mosquitoes: “It’s useless, they should look for another method, another way” (RFGD). Participants also indicated that the insecticide used is not effective: “when the mosquito detects the spray they go to their breeding site [...] and as soon as the fumigation is over the [mosquito] goes back to where it has to go to bother us” (RFGD).

From another perspective, the opinions mentioned in the reluctant group were related to sensory experiences and to the perception that fumigation affects their senses, especially smell. “In the first place, the smell is too strong, too invasive, too horrible.” (RFGD). In the accepting focus group discussion (AFGD), we investigated the same categories previously discussed by the RFGD. Participants with previous experience with fumigation were generally satisfied with the treatment, as they were able to notice the results: “When they come to my house, I am happy, because I leave my house closed for one or two hours, I close windows and doors to make sure it works” (AFGD).

Although participants of the AFGD were satisfied with the treatment, when asked about suggestions to improve the program, one issue that emerged was the need to receive more and better information. “Because there are so many doubts, people first need to be taught what is going to happen” (AFGD). Similar to the RFGD, the participants of the AFGD also suggested including green areas and streams in the treatments. “Even when they went to my house, I said: ‘Fumigate the stream; that is where most mosquitoes are’” (AFGD). Another participant said: “I think it is best to fumigate the stream first, and then the rooms and houses, so these animals stay there in the stream” (AFGD).

Focus group discussion participants view fumigation as a method of mosquito control. In addition, they perceive that it is a method through which other pests can be controlled. However, FGD participants also argued that both mosquitoes and other pests come from environments outside of households that were not treated. Households are perceived as safe places where the reproduction of the vector does not occur, and for this reason, they recommend treatment of spaces outside of dwellings.

## Discussion

The acceptance of fumigation treatment in the houses of this study was 55.0%, substantially lower than targets set by the ministries of health of other Latin American countries, where a minimum acceptability of 95% is expected [19]. However, more than 80.0% of survey participants deemed fumigation useful, many of which also recognized the potential for fumigation to kill other non-target insect pests. In a comparable study examining attitudes towards long-lasting insecticidal net screens, similar perceptions of efficacy were reported; 90.0% of participants noted decreased density of mosquitoes [33]. Thus, the perception that fumigation by the SSM lacks efficacy is not a considerable problem.

The main barrier to treatment with fumigation observed in this study was the high proportion of closed houses at the time of the visit, resulting in below-target coverage. Palma-Pinedo [18] reported that one of the main problems in vector control is that scheduled treatments do not conform to the routines of families. Particularly, residents who work far from home can’t be present when vector control activities normally take place [34, 35]. As SSM fumigation is performed on weekdays and during working hours, it is possible that the proportion of closed houses observed in this study can be explained by this factor [18, 35]. Health officials should consider this barrier and provide a second opportunity for treatment during non-business hours.

The primary reason for people who rejected the treatment with fumigation was being busy at the time of treatment (33.1%). This parallels a report by Buttenheim et al. [36], in which a high percentage of respondents did not allow the treatment due to lack of time or because they had to leave their home to work. Another reason for hesitance in this study was a lack of interest in the treatment (24.5%). Paz-Soldán et al. [35] observed that 11.0% of participants did not consider the treatment necessary; they argued that lack of coverage could be associated with a low perceived effectiveness of the treatments since some people use their own methods of pest control. Such a mentality may help to explain the findings observed in the present study.

Rejection of fumigation was also commonly attributed to the perception that fumigation negatively impacts health. Adjusted for other variables, residents that expressed concern over the effect of fumigation on human or animal health were twice as likely to refuse treatment than those without such concerns. Consistently, FGD also raised this worry. Members of FGD noted that respiratory allergies and headaches were the most frequent side effects of fumigation, which is in agreement with reports of other studies [35, 37]. A high proportion of survey participants in our study (67.6%) also thought that the odor of the insecticide is unpleasant—a quality that FGD members associated with detrimental health effects. Paz-Soldán et al. [35] similarly reported both a foul odor and health concerns as reasons for rejection of fumigation. Thus, an alternative, better-smelling formulation of the insecticide may increase participation in fumigation campaigns as well as alleviate concerns of unpleasant side effects.

While more than 80% believed fumigation was useful, lack of trust in its efficacy nonetheless contributed to some noncompliance. One reason for the doubtful attitude towards fumigation was the observation of mosquitoes immediately after treatment. Palma-Pinedo et al. [18] noted similar concerns in their study, with participants claiming that mosquitoes leave homes during treatment but return soon after. Such lack of perceived utility may be attributed to the fact that the insecticide used has no residual effect and only acts while suspended in the air [10].

Another reason that residents may deem fumigation ineffective is their association of mosquitoes with certain outdoor habitats. Participants in both focus groups discussed that mosquitoes and other pests proliferate in untouched outdoor spaces, such as un-channeled streams, vacant lots, recycling sites, and garbage dumps, and that mosquitoes colonize homes from these places. Similar perceptions were noted by Palma-Pinedo in Peru, in which it was argued that mosquitoes originate in fields and abandoned houses and from there colonize dwellings [18]. Thus, the association of mosquitoes with outdoor habitat deflates confidence in a control measure that focuses on indoor spaces.

In addition to social barriers to treatment, we observed that fumigation was usually conducted well after the target timeframe had passed. Given that the period of infectivity of a person in a viremic state is less than 1 week [2, 24], and that mosquitoes that may have bitten this person require roughly 9 days to become infectious at ambient temperatures [22], the interval between notification and treatment should not exceed this extrinsic incubation period. Therefore, for interventions to be carried out in such a timely manner, it is imperative that both epidemiological surveillance systems and the implementation of vector control activities are streamlined.

In the present study, the following methodological limitations are recognized. In the first place, it was not possible to perform a random selection of the neighborhoods because only those that were treated by the SSM during the study period were included. On the other hand, memory bias on the part of the beneficiaries could have generated erroneous answers when answering the questionnaire. Finally, not all the information registered in the SIVIGILA Was complete and could be verified, which is why it was not possible to directly contact the index cases, which significantly reduced the sample size to determine the opportunity for treatment.

## Conclusion

In this study, we identify barriers that limit thermal fogging coverage in Medellin, as well as attitudes and beliefs that reduce acceptance of fumigation as a mosquito control intervention. The limited coverage of thermal fogging restricts adequate control of the vector, and therefore the reduction of the dengue transmission is hindered. The most common reason for not fumigating a home was that no one was home at the time of visiting. When residents were home, the most common reasons for rejection were the perception that fumigation affects health or is unnecessary (due to lack of mosquitoes in the home). Given that total fumigation coverage in Medellin was below target, we suggest that fumigation campaigns establish a system to increase coverage, varying the time at which they visit homes and providing better and more information regarding the treatment to residents. Furthermore, we identified a substantial lag between notification of the epidemiological surveillance system and fumigation. This system should be streamlined so that treatments can, ideally, kill potentially infectious mosquitoes before their extrinsic incubation period has passed.

## Additional files

Additional file 1: Questionnaire. Format used to collate information necessary to development the research. (PDF 516 kb)

Additional file 2: Flowchart of included cases. Flowchart detailing exclusion criteria for dengue cases in the analysis of treatment timing. (JPG 61 kb)

Additional file 3: Database. Results of the variables included in the study. (XLSX 287 kb)

## Data Availability

All data generated or analyzed during this study are included in this published article [Additional file 1 Questionnaire, Additional file 3 Database].

## Abbreviations

AFGD: Accepting Focus Group Discussion
CI: Confidence Interval
FGD: Focal Groups Discussion
IRQ: Interquartile Range
OR: Odds Ratio
RFGD: Reluctant Focus Group Discussion
SIVIGILA: Epidemiological Surveillance System
SSM: Secretary of Health of Medellin
WHO: World Health Organization
