# Supplementary material for: Barriers that limit the implementation of thermal fogging for the control of dengue in Colombia: a study of mixed methods
Source: BMC Public Health. 2019 May 30;19:669. doi: 10.1186/s12889-019-7029-1 (PMC6543676; doi:10.1186/s12889-019-7029-1)
Supplement: Supplementary file 1 — Questionnaire. Format used to collate information necessary to development the research. (PDF 516 kb) [file 12889_2019_7029_MOESM1_ESM.pdf]

UNIVERSIDAD DE ANTIOQUIA

Barriers that limit the implementation of thermal fogging for the control of dengue in Colombia

QUESTIONNAIRE

CONFIDENTIAL

The information contained in this questionnaire is confidential, it will only be used for research purposes and the results will be presented anonymously

Code ____

1 Date D D M M Y Y

2 Neighborhood ____ **3 Commune** ____

PERSONAL INFORMATION

4 Full name ____

5 Date of birth D D M M Y Y

6 Address ____

7 Telephone number ____ **8 Cell phone** ____

9 Gender Man [1] Woman [0]

10 Educational level achieved

None	1	High school incomplete	4	Higher education	7
Elementary incomplete	2	High school	5	Postgraduate	8
Elementary complete	3	Higher education incomplete	6		

11 What is your occupation?

Housewife	1	Employee	3	unemployed	5
Student	2	Informal	4	retired	6

12 Does anyone of legal age stay at home?

All the time	1	Only at the afternoon	3	Only in the night	4
Only in the morning	2				

13 How long have you lived in this neighborhood? Years ____ Months ____

SENSITIZATION

		Yes	No
14	Did the official identify himself?	1	0
15	Was the official polite?	1	0
16	Did the official say there are cases of dengue in the neighborhood?	1	0
17	Did the official explain clearly why fumigation is necessary?	1	0
18	Did the official explain the procedure to be performed?	1	0

THERMAL FOGGING

19 Did you accept that the fumigation was carried out inside the dwelling? **Yes** 1 **No** 0

20 Do you think fumigation is beneficial to prevent dengue? **Yes** 1 **No** 0

21 Why?

22 I will read a statement about the fumigation and you will answer Yes o Not according your consideration

	Y	N	NK		Y	N	NK
Kill mosquitoes	1	0	2	Affects the people's health	1	0	2
Control pest	1	0	2	Affects pets' Health	1	0	2
Protects from dengue	1	0	2	Damage furniture in the house	1	0	2
It's free	1	0	2	Causes bad smell	1	0	2
The officials generate trust	1	0	2	Harm the environment	1	0	2

23 Which does institution do the fumigation?

Secretary of Health of Medellin	1	Private company	3	Other, which	5
firemen	2	Do not know	4		

24 Has the house been visited previously to be fumigate whit thermal-fogging?

Yes 1 No 2 Do not remember 3

25 When was the last time that the house was fumigated with thermal-fogging?

Not applicable	1	Between 7-12 months	4	More than two years	6
Less than three months	2	Between 1-2 years	5	Do not remember	7
Between 3-6 months	3				

26 How many times in the last year?

None	1	twice	3	More than three times	5
Once	2	Three times	4	Do not remember	6

27 When the officials of Secretary of Health come to perform the fumigation with thermal-fogging Do you accept?

Does not apply	1	sometimes	3
always	2	Never	4

KNOWLEDGE AND PRACTICES

28 How is dengue transmitted?

Eating contaminated food and/or drinking contaminated water	1	Contact with a sick person	5
For living near to streams	2	Garbage around the house	6
Lack of cleanliness at home	3	Do not know	7
Mosquito bite	4	Other? which	8

29 Where does mosquitoes that transmit dengue breed?

Do not apply	1	Stagnant clear water	4	Do not know	7
Stagnant dirty water	2	trees	5	Other? which	8
Dumpsters	3	Streams	6		

30 What symptoms do you associate with dengue?

fever	1	Headache	5	Hemorrhage	9
sickness/threw up	2	rash	6	Other? which	10
Muscle and joint pain	3	weakness	7		
Diarrhea	4	retro-ocular pain	8		

31 Do you think dengue disease to be?

Little severe	1	Very severe	3	Do not know	4
Moderately severe	2				

32 Have you, a relative or someone close to you been diagnosed with dengue?

**Yes**	1
**No**	0

33 How long ago did you or your relative diagnose with dengue?

Do not apply	1	Between 7-12 months	4	More than two years	6
Less than three months	2	Between 1-2 years	5	Do not remember	7
Between 3-6 months	3				

34 What precautions do you take to prevent dengue?

	Y	N	N.A.		Y	N	N.A.
Use insecticides to fumigate the house	1	0	2	Cover water containers	1	0	2
Eliminates water deposits	1	0	2	Participates in SSM's campaigns	1	0	2
Wash water deposits	1	0	2	Other? which	1	0	2
Use repellents	1	0	2				
Close doors and windows	1	0	2				

HOUSING INFORMATION

35 Number of inhabitants in the dwelling

36 How many rooms does the house have (living room included)?

37 Socioeconomic stratum

38 How do you get water for the house?

Aqueduct EPM	1	Rural aqueduct	4	Other? which	6
rainwater	2	Birth of water/waterhole	5		
neighbors	3				

39 Do you have a pet? **Yes** 1 **No** 0

40 What kind of pet?

Do not apply	1	Cat	3	birds	5
Dog	2	Fishes	4	Other? which	6

41 Do you have containers without cover that can store water?

Yes	1
No	0

42 What kind of containers

	Yes	No		Yes	No
Do not apply	1	0	Tank	1	0

Buckets	1	0	Bottles	1	0
Cans	1	0	Other? which	1	0
Flower vase	1	0			

43 Have you felt or seen mosquitoes inside home? **Yes** 1 **No** 0

Official who carries out the sensitization

filled by:
