# Supplementary figures and images for: Barriers that limit the implementation of thermal fogging for the control of dengue in Colombia: a study of mixed methods
Source: BMC Public Health. 2019 May 30;19:669. doi: 10.1186/s12889-019-7029-1 (PMC6543676; doi:10.1186/s12889-019-7029-1)

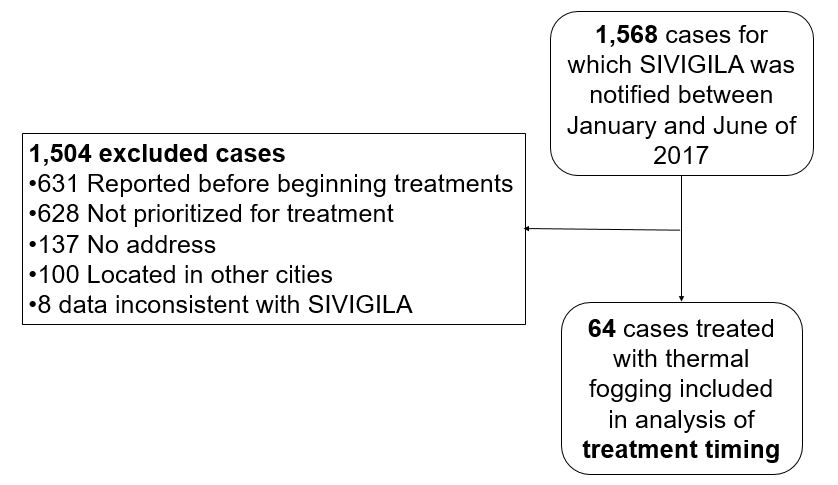

Supplement: Supplementary file 2 — Flowchart of included cases. Flowchart detailing exclusion criteria for dengue cases in the analysis of treatment timing. (JPG 61 kb) [file 12889_2019_7029_MOESM2_ESM.jpg]
